# Barriers for nurses’ participation in and utilisation of clinical research in three hospitals within the Kumasi Metropolis, Ghana

**DOI:** 10.11604/pamj.2018.30.24.15230

**Published:** 2018-05-15

**Authors:** Isaac Nkrumah, Catherine Atuhaire, Gunilla Priebe, Samuel Nambile Cumber

**Affiliations:** 1 Department of Nursing, University of Buea, Cameroon; 2 Kwame Nkrumah University of Science and Technology Hospital, Ghana & Garden City University College, Ghana; 3 Faculty of medicine, Department of Nursing, Mbarara University of Science and Technology, Uganda; 4 Section for Epidemiology and Social Medicine, Department of Public Health and Community Medicine, Institute of Medicine (EPSO), The Sahlgrenska Academy at University of Gothenburg, Box 453, SE-405 30 Gothenburg, Sweden

**Keywords:** Evidence based medicine, nurses´ professional role, delivery of health care, clinical nursing research, Ghana health care sector

## Abstract

**Introduction:**

Scientific research results are a necessary base for high quality nursing practice. The level of implementation of research in the clinical setting, including nurses' participation in and knowledge of research results, have gained considerable attention internationally. However, the remarkable international increase of such studies does not apply to the Ghanaian context. We therefore set out to evaluate the degree of involvement of nurses in research, as well as their utilization patterns of research findings in Ghana. Objectives: the study sought to investigate the proportion of nurses involved in clinical research as well as barriers for nurses' participation and utilization of research findings, within the Kumasi Metropolis area, Ghana.

**Methods:**

A descriptive and analytical cross-sectional study design was used in this study. A 47 item questionnaire elicited data on 158 participants' demographics, the proportion and the barriers to participation, which was then analyzed using SSPS version 17 software. Qualitative interviews with key informants complemented quantitative survey data. In-depth interviews with nurse managers at the hospitals in focus was conducted and analyzed thematically.

**Results:**

The study shows that 36.1% of the nurses, included in the study, had participated in research and only 25.3% of these had (knowingly) used specific research results in clinical practice. However, the level of research participation differed greatly between nurses working at different hospitals. Nurses' participation in clinical research was shown to be associated with their perception of benefit of research to practice as well as their understanding of time as a factor for them engaging in reading scientific articles. In addition, barriers associated with nurses' integration of research findings into the daily practice was their perceived lack of support from the collegium and their perception of research as not part of the nursing role.

**Conclusion:**

Findings from the study suggest that there is a need to create institutional support to facilitate and encourage nurses' participation in research, yet also to formalize nurses' continuous professional development. This, could change nurses' attitudes towards research, and contribute to improving health care as it would increase nurses' role as agents for evidence based clinical practice.

## Introduction

The need for health care staff to appreciate and engage with scientific evidence cannot be underestimated. Even though it cannot wholly replace the so called “clinical eye”, i.e experience based knowledge gained from working in a clinical setting, tradition and homegrown trial-and-error cannot be the main source of knowledge for 21^st^century high quality health care [1,2]. Nursing research, which is defined as a systematic inquiry designed to develop knowledge about issues of importance to nurses, including nursing practice, nursing education, and nursing administration [3,4]. It is directed towards promoting professional quality, ie. the credibility, accountability and cost effectiveness of nursing care [2]. The American Association of Colleges of Nursing (AACN) has described nursing research as part of the field “clinical research” which attends to issues such as acute to chronic care experiences throughout life, health promotion and preventive care to end-of-life care; and care for individuals, families and communities in diverse settings [5]. Nursing research thus also promotes evidence based nursing practice and it should therefore be as imperative to nurses as biomedical research is to eg. medical doctors [2, 3]. Medical practice, including nursing, has evolved a great deal since the mid-19^th^century when Florence Nightingale laid the foundation for professional nursing and the first school of nursing was established [5]. In the aspect of research, nursing practice has been in the focus for a century, yet did more recently become a scientific discipline in its own right. Although advances have been made, the larger portion of nurses with direct research involvement is seen primarily in academia while it is rare for direct care nurses, working outside of university hospitals, to engage in research activities and thus cultivate a culture of research [6-9]. Owing to this, nursing research still, to a large degree, remains a university topic carried out within and by academia. Several studies on the factors that hinder nurses' participation and utilization of research identified lack of time on the job, lack of knowledge, lack of funding, nurses poor attitude/perception towards research and lack of research support services as the key constraints [7,8, 10-14 ]. Some studies within this field have been carried out in Nigeria [13, 14], but little is known about clinical nurses' involvement in research, in Ghana. In addition, despite some efforts by the Nursing and Midwifery Council of Ghana, very little is seen from graduates of the various nursing programs working in clinical settings regarding clinical research. In order to gain a more precise understanding of the situation this study sought to assess the involvement and utilization of clinical research by direct care nurses. The overarching aim of the study is to identify strategies to improve the scientific quality of nursing practice and “consequently” patient care. Specifically, the research question in focus of the study was how many clinical nurses participate and utilize research findings in three hospitals within the Kumasi Metropolis was addressed? Moreover, the question of what barriers and socio-demographic factors influence participation and utilization in clinical research among these nurses were posed.

## Methods

A descriptive and analytical cross-sectional quantitative design, with complementary qualitative interviews, was used in carrying out the study among 158 clinical nurses in three hospitals within the Kumasi Metropolis from October 2014 to June 2015. The hospitals Komfo Anokye Teaching Hospital (KATH); directorate of Surgery and directorate of Accident & Emergency Medicine, Kumasi South Government Hospital and the Kwame Nkrumah University of Science and Technology (KNUST) Hospital was selected as these are responsible for teaching and represents the different levels of healthcare delivery in Ghana. Moreover, these hospitals have all the categories of nurses with different levels of educational qualifications hence different experiences and research exposure. The inclusion of these hospitals was done in accordance with purposeful sampling logic, ie. it aimed at generating varied views related to the study title across all the levels of healthcare delivery within the country. However, all involved hospitals are located in one region, out of the ten regions in the country. Inclusion criteria for participants were involvement in clinical practice, a minimum of a diploma nursing qualification and consent to be part of the study. Excluded from the study were part-time nurses, nurses on leave and nurses with a less than six months clinical experience. A convenient sampling method was used to select the participants involved in the study. To determine the sample size for the study, the formula below was used.

$$n=\frac{Z^{2}pq}{d^{2}}$$

z = standard normal deviate; =1.96, p = since no previous study has been conducted in this setting, a pre-study estimate of the proportion of nurses involved in clinical research; = 0.5 (50%), q = 1- p; p= 1-0.5= 0.5, d = amount of error to be allowed; = 0.05 (5%)

$$n_{0}=\frac{{\left(1.96\right)}^{2}\times 0.5\times 0.5}{{\left(0.5\right)}^{2}}$$

$$n_{0}=\frac{0.9604}{0.0025}$$

Sample size for an infinite population, n_0_ = 384 clinical nurses However, due to the limited time and inadequate time for the study, a finite population correction was done using;

$$n=\frac{n_{0}}{\left(1+n_{0}/N\right)}$$

Where: n = sample size for the finite population of clinical nurses within the three selected hospitals n_0_ = assumed sample size for an infinite population of clinical nurses N = Size of the population of nurses who met the inclusion criteria for the study within the three selected hospitals; N_1_ + N_2_+ N_3_ Sample size for the finite population for the study, n = 214 clinical nurses A total of 214 questionnaires were therefore administered, out of which 158 were completed, representing a 74% response rate. A close-ended questionnaire, with a four category Likert scale, based on the Research Utilization BARRIER Scale was used [15]. However, the items in the questionnaire were modified in order to better fit the study objectives and context. The modification was done in response to a Pre-test carried out at similar hospitals (compared to those who was later included in the study). The questionnaire and the interview guide (see below) were also reviewed by an Msc Nursing graduate, a PhD Nursing student as well as my local collaborator, and the data collection instruments were amended accordingly. The questionnaires were administered to the nurses on their various wards/unit during their usual schedules, after written consent had been obtained. The items on the Research Utilization BARRIER Scale are grouped under four categories: “Characteristics of the Setting”, “Presentation of research”, “Qualities of the Nurse” and “the Characteristics of the Research”. Yet, only “Characteristics of the setting” and “Qualities of the nurse” were considered in this study. In addition, data on sociodemographic factors were obtained. Quantitative data was analyzed using SPSS version 17.0. Both univariate and bivariate analysis were done. Thus to determine the number of nurses who had ever participated in clinical research after school, frequencies and proportions of use was calculated. The same was done in determining those who had ever used recommendation from any research (globally or locally) to change their practice. The barriers to both the participation and use of research findings/recommendations on the job were therefore determined. A chi-square test was used to determine the association between the socio-demographic factors and nurses' participation and utilization of clinical research. Level of significance was also set at a P-value of 5%. As a complement to the questionnaires, interviews with key informants-nurse managers working at three of the included hospitals-were carried out. Answers to each question in the interview guide were written down by the informants as they declined tape recording of the interviews. A thematic analysis was then done, relating the interviews to the research questions.

**Ethical considerations:** Ethical approval was obtained from the Committee on Human Research, Publication and Ethics (CHRPE), School of Medical Sciences, KNUST. However, administrative approvals were obtained from the administrations of the three selected hospitals. Data collection process began after approval had been granted by the Committee on Human Research, Publication and Ethics (CHRPE).

## Results

**Distribution of participants by demographic factors**: As presented in Table 1, out of the 158 nurses, 92 (58.2%) were females. With regards to their educational qualification, 92 (58.2%) were diploma holders, yet only 1 (0.6%) had a master's degree and the remaining had a first degree in nursing. The mean age and years of practice among the participants were 29.02 ± 2.34 and 3.23 ± 1.76 years respectively.

**Table 1:** Distribution of participants by demographic factors

Demographic factor	Frequency (n)	Proportion (%)
**Gender**		
Male	66	41.8
Female	92	58.2
**Educational Qualification**		
Diploma	92	58.2
Degree	65	41.1
Masters	1	0.6
**Speciality in Nursing**		
General	143	90.5
Education	2	1.3
Other	13	8.2
**Rank**		
Staff Nurse	41	25.9
Senior Staff Nurse	55	34.8
Nursing Officer	42	26.6
Senior Nursing Officer	19	12.0
Other	1	0.6
**Years of Practice**		
Mean (Standard deviation, SD)	3.23 (1.76)	
Minimum	1.00	
Maximum	15.00	
**Age**		
Mean (Standard deviation, SD)	29.02 (2.34)	
Minimum	24.00	
Maximum	45.00	

**Proportion of nurses who have ever participated clinical research**: Out of the 158 participants, 57 (36.1%) had been involved in nursing research (after graduating from nursing school), while 101 (63.9%) had not. Out of the 57 nurses who had ever participated in a clinical research (after school), only 3 (1.9%) were Principal investigators of those studies carried out while 43 (27.2%) were study participants and 11 (7.0%) were data collectors. The level of participation by the nurses in clinical research differed greatly among the various hospitals. While 30 (70.2%), of the 57 who had ever participated in research, were from KATH (Surgery and A&E), only 17 (29.8%) were from the Kumasi South and 10 (17.5%) from KNUST Hospitals.

### Barriers influencing nurses participation in clinical research

**Association between characteristics of the setting and nurses participation in clinical research**: As shown in Table 2, the majority 124 (78.5%) of the participants perceived lack of funding to be a barrier for participation in research, while 14 (8.9%) disagreed and 20 (12.7%) were neutral. Moreover, 90 (57%) of the study participants pointed out lack of support and approval from hospital authorities as a barrier, while 32 (20.3%) disagreed, and 36 (22.8%) were neutral to this possible barrier. As also indicated in Table 2, 89 (56.3%) agreed that absence of research committee in hospital was a barrier, 42 (26.6%) disagreed while 27 (17.1%) were neutral. Ninety (57%) of the 158 participants agreed that insufficient time on the job was a barrier to conduct research, 41 (25.9%) disagreed and 27 (17.1%) were neutral. In determining the association between the variables and participation in research, only insufficient time on the job to read research articles was found to be statistically significant as 16 (25.8%) with a P-value of 0.04 of the 62 nurses who agreed had ever participated in research after school Table 2.

**Table 2:** Association between characteristics of the setting and nurses participation in clinical research

Characteristics of the setting	Number of participants in each category of predictor (N)	Frequency of participants who participate in clinical research (n)	Proportion of participants who participate in clinical research (%)	OR (95% CI)	P-value
**a) Lack of funding**					
Disagree	14	4	28.6	-	-
Agree	124	49	39.5	1.63 (0.49-5.50)	0.43
Neutral	20	4	20.0	0.63 (0.13-3.08)	0.56
**b) Lack of support from hospital authorities**					
Disagree	32	9	28.1	-	-
Agree	90	37	41.1	1.78 (0.74-4.29)	0.20
Neutral	36	11	30.6	1.12 (0.39-3.20)	0.83
**c) Lack of reward by authorities**					
Disagree	26	13	50.0	-	-
Agree	103	36	35.0	0.54 (0.25-1.28)	0.16
Neutral	29	8	27.6	0.38 (0.12-1.18)	0.09
**d) Absence of research committee in hospital**					
Disagree	42	13	31.0	-	
Agree	89	32	36.0	0.25 (0.57-2.74)	0.57
Neutral	27	12	44.4	1.78 (0.66-4.86)	0.26
**e) Lack of support from colleagues**					
Disagree	45	17	37.8	-	-
Agree	80	31	38.8	1.04 (0.49-2.21)	0.91
Neutral	33	9	27.3	0.62 (0.23-1.64)	0.33
**f) Insufficient time job to do research**					
Disagree	41	13	31.7	-	-
Agree	90	32	35.6	1.19 (0.54-2.61)	0.67
Neutral	27	12	44.4	1.72 (0.63-4.70)	0.29
**g) Insufficient time on job to read articles**					
Disagree	59	26	44.1	-	-
Agree	62	16	25.8	0.44 (0.21-0.95)	**0.04**
Neutral	37	15	40.6	0.87 (0.38-1.99)	0.73

**Association between qualities of the nurse and participation in clinical research**: As presented in Table 3, of the 158 nurses, 75 (47.5%) agreed that nurses' perception about research as not nursing role was a barrier, 67 (42.4%) disagreed and 16 (10.1%) were neutral. Moreover, while 50 (31.6%) agreed that lack of knowledge to conduct research was a barrier, 71 (44.9%) disagreed and 37 (23.4%) were neutral. Nurses' perception about research as not a nursing role was found to be associated with participation in clinical research as 9 (56.3%) with a P-value 0.04 of the 16 who were neutral had participated in research after their nursing education Table 3. Four interviews were conducted with nurse managers, who pointed out lack of time, lack of funding and nurses poor perception about research to be barriers influencing research participation among nurses. *Nurse Manager 1: “research is good but the workload is too much and because of that it is difficult getting time to even think of research” Nurse Manager 2: “the staff are few but we have plenty patients to attend to, so there's limited time to do research. Besides I don't think the nurses are willing to use their money to do that. Some nurses don't even see it as part of their duties” Nurse Manager 3: “as a nurse, obviously research is very important but it requires lot of money and also we don't have time to engage in it. Nurse Manager 4: “most of we nurses, our focus is to work and get our salaries. Research is a secondary matter. The busy nature of our work also prevents us”*

**Table 3:** Association between qualities of the nurse and participation in clinical research

Qualities of the Nurse	Number of participants in each category of predictor (N)	Frequency of participants who participate in clinical research (n)	Proportion of participants who participate in clinical research (%)	OR (95% CI)	P-value
**a) Insufficient knowledge in writing research proposal**					
Disagree	62	21	33.9	-	-
Agree	72	28	38.9	1.24 (0.61-2.52)	0.55
Neutral	24	8	33.3	0.98 (0.36-2.65)	0.96
**b) Research perceived as not a nursing role**					
Disagree	67	19	28.4	-	-
Agree	75	29	38.7	1.59 (0.79-3.23)	0.20
Neutral	16	9	56.3	3.25 (1.06-9.97)	**0.04**
**c) Lack of Knowledge to do research**					
Disagree	71	23	32.4	-	-
Agree	50	20	40.0	1.39 (0.66-2.95)	0.39
Neutral	37	14	37.8	1.27 (0.55-2.91)	0.57

**Association between socio-demographic factors and nurses participation in clinical research**: The factors considered in this study were: nurses' educational qualification, years of practice, gender and rank. As shown in Table 4, 23 (25%) of 92 diploma nurses had ever participated in clinical research, 33 (50.8%) of the 65 degree nurses had participated and 1 (100%) with masters had participated. Educational qualification of nurses was found to be associated with participation in clinical research as it recorded a P-value of 0.02. Nurses' years of practice, gender and rank in the profession were however not found to be associated with participation in clinical research Table 4.

**Table 4:** Association between socio-demographic factors and nurses participation in clinical research

Factors	Number of Nurses in each category of Predictor	Frequency of nurses who participate in Research (n)	Proportion who participate in research (%)	P value
**Educational Qualification**				
Diploma	92	23	25.0	0.02
Degree	65	33	50.8	
Masters	1	1	100	
**Years of Nursing Practice**				
1-5	150	53	35.3	0.38
6-10	7	4	57.1	
11-15	0	0	0	
**Gender**				
Male	66	25	37.9	0.69
Female	92	32	34.8	
**Rank**				
Staff Nurse	41	9	22.0	0.19
Senior Staff Nurse	55	23	41.8	
Nursing Officer	42	17	40.5	
Above Nursing Officer	20	8	40.0	

**Proportion of nurses who had ever used clinical research**: The study participants were also asked to indicate whether they had ever used recommendation from any study globally in their practice after nursing school. It was found that only 40 (25.3%) of the 158 nurses had ever used a recommendation from research to change their practice.

### Barriers influencing the use of clinical research by nurses

**Characteristics of the setting and use of clinical research among nurses**: As shown in Table 5, while 86 (54.4%) of the 158 nurses agreed that inadequate facilities for implementation of research findings in their setting was a barrier, 37 (23.4%) disagreed and 35 (22.2%) were neutral. Most of the nurses 95 (60.1%) agreed that minimal authority on their part to change procedure was a barrier, 41 (25.9%) disagreed and 22 (13.9%) were neutral. However, only 60 (38%) of the participants agreed that insufficient time on the job to implement new ideas was a barrier, while 71 (44.9%) disagreed 27 (17.1%) were neutral. However, a bivariate analysis of the characteristics/variables under the setting in which nurses practice revealed that of the 77 nurses who agreed that lack of cooperation from physicians and colleagues was a barrier, 20 (26.0%) with a P value of 0.03 had ever used research in their practice. Lack of cooperation was therefore the only statistically significant variable Table 5.

**Table 5:** Association between characteristics of the setting and use of clinical research by nurses

Characteristics of the Setting	Number of nurses in each category of Predictor (N)	Frequency of nurses who use Clinical Research (n)	Proportion of nurses who use Clinical Research (%)	OR (95% CI)	P-value
**a) Inadequate facilities for implementation**					
Disagree	37	10	27.0	-	
Agree	86	20	23.3	0.82 (0.34-1.98)	0.66
Neutral	35	10	28.6	1.08 (0.38-3.03)	0.88
**b) Lack of time to read research**					
Disagree	69	14	20.3	-	
Agree	60	17	28.3	0.55 (0.69-3.50)	0.29
Neutral	29	9	31.0	1.77 (0.66-4.72)	0.26
**c) Perceived minimal authority to change procedure**					
Disagree	41	8	19.5	-	-
Agree	95	28	29.5	1.72 (0.71-4.20)	0.23
Neutral	22	4	18.2	0.92 (0.24-3.46)	0.90
**d) Lack of cooperation from physicians and colleagues**					
Disagree	48	6	12.5	-	
Agree	77	20	26.0	2.46 (0.91-6.65)	0.08
Neutral	33	14	42.4	5.16 (1.72-15.48)	**0.003**
**e) Lack of support from administration**					
Disagree	52	10	19.2	-	
Agree	59	16	27.1	1.56 (0.64-3.83)	0.33
Neutral	47	14	29.8	5.16 (1.72-15.48)	0.22
**f) Insufficient time to implement new ideas**					
Disagree	71	15	21.1		
Agree	60	17	28.3	1.48 (0.66-3.28)	0.34
Neutral	27	8	29.6	1.57 (0.58-4.29)	0.38

**Qualities of the nurse influencing use of clinical research in the clinic**: As presented in Table 6, when participants were asked of their perception about the benefit of research on their practice as a barrier, less than half 68 (43.0%) agreed it was beneficial, 47 (29.7%) disagreed and 43 (27.2%) were neutral. Moreover, while 77 (48.7%) agreed to lack of generalizable research to their particular setting as a barrier, 35 (22.2%) disagreed while 46 (29.1%) were neutral. Lastly, of the 158 participants, 76 (48.1%) agreed that nurses unwillingness to change was a barrier to research utilization, 66 (41.8%) disagreed while 16 (10.1%) were neutral. A bivariate analysis of the variables revealed that only perceived minimal benefit of research on practice and nurses' unwillingness to change were statistically significant with P values of 0.02 and 0.01 respectively Table 6. The qualitative interviews again revealed similar tendencies as listed in the “Characteristics of the setting” and “Qualities of the nurse”. Perceived minimal authority to change; inadequate facilities for implementation; results not generalizable to own setting and nurses' lack of enthusiasm for change were pointed out by the key-informants as the barriers influencing use of research findings among nurses. *Nurse Manager 1: “research findings are useful but we as nurses do not have much power to change things” Nurse Manager 2: “sometimes you want to apply the new things from research but we do not have the needed equipment to implement some of the recommendations and our hospital set up also makes it difficult” Nurse Manager 3: “some nurses, especially the old ones are used to doing things the old ways and not ready to embrace the new trend”. Nurse Manager 4: “from my experience, most nurses are comfortable doing things the old way”*

**Table 6:** Association between qualities of the nurse and use of research in practice

Qualities Of The Nurse	Number of nurses in each category of the Predictor (N)	Frequency of nurses who use Clinical Research (n)	Proportion of nurses who use Clinical Research (%)	OR (95% CI)	P-value
**1)Ignorance about the research**					
Disagree	70	18	25.7	-	-
Agree	51	14	27.5	1.09 (0.48-2.47)	0.83
Neutral	37	8	21.6	0.80 (0.31-2.06)	0.64
**2) Perceived minimal benefit on practice**					
Disagree	47	6	12.8	-	-
Agree	68	23	33.8	3.49 (1.29-9.43)	**0.01**
Neutral	43	11	25.6	2.35 (0.78-7.03)	0.13
**3)Results not generalizable to own setting**					
Disagree	35	9	26	-	-
Agree	77	18	59	0.88 (0.35-2.22)	0.79
Neutral	46	13	33	1.14 (0.42-3.07)	0.80
**4) Isolation from knowledgeable colleagues to discuss research**					
Disagree	62	18	29.0	-	-
Agree	62	10	16.1	0.47 (0.20-1.12)	0.09
Neutral	34	12	35.3	1.33 (0.55-3.25)	0.52
**5) No value for research**					
Disagree	86	22	25.6	-	-
Agree	47	14	29.8	1.23 (0.56-2.72)	0.60
Neutral	25	4	16.0	0.55 (0.17-1.79)	0.32
**6) Incapable of evaluating research quality**					
Disagree	66	14	21.2	-	-
Agree	26	18	27.3	1.39 (0.63-3.10)	0.42
Neutral	66	8	30.8	1.65 (0.59-4.58)	0.34
**7) No documented need to change practice**					
Disagree	60	14	23.3	-	-
Agree	59	12	20.3	0.84 (0.35-2.01)	0.69
Neutral	39	14	35.9	1.84 (0.76-4.47	0.18
**8) Unwillingness to change**					
Disagree	66	14	21.2	-	-
Agree	76	18	23.7	1.15 (0.52-2.55)	0.73
Neutral	16	8	50.0	3.71(1.18-11.66)	**0.02**

**Socio-demographic factors influencing utilisation of clinical research by nurses**: The demographic factors considered in this study include educational qualification, years of nursing practice, gender and rank. In determining the association between these variables and utilization of research by nurses, none of them was found to be statistically significant. As shown in Table 7, educational qualification, years of practice, gender and rank recorded P-values of 0.08, 0.12, 0.40 and 0.24 respectively Table 7.

**Table 7:** Association between socio-demographic factors and use of clinical research in practice

Factors	Number of Nurses in each category of Predictor	Frequency of nurses who use Research in Practice (n)	Proportion who use research in Practice (%)	P value
**Educational Qualification**				
Diploma	92	19	20.7	0.08
Degree	65	20	30.8	
Masters	1	1	100	
**Years of Nursing Practice**				
1-5	150	36	24.0	0.12
6-10	7	3	42.9	
11-15	1	1	100	
**Gender**				
Male	66	19	28.8	0.40
Female	92	21	22.8	
**Rank**				
Staff Nurse	41	13	31.7	0.24
Senior Staff Nurse	55	9	16.4	
Nursing Officer	42	11	26.2	
Above Nursing Officer	20	7	35.0	

## Discussion

**Proportion of nurses who have ever participated and used clinical research**: The study shows that less than half (36.1%) of the nurses who participated in the study had ever been involved in a nursing research. It also shows that nurses, when they do participate, the absolute majority of them do it as study participants, ie. not as active researchers. A cause, or effect, of this was that only a few perceived research as part of the nursing role. These findings are consistent with similar studies in China [16] and Nigeria [13, 14]. This is troublesome, as nursing research forms the key foundation for nursing practice and a minimal research participation by nurses therefore risks affecting patient care adversely. The result regarding nurses ever using a research finding to change their practice showed that only 40 (25.3%) of the 158 participants had done so, after nursing school. This too corresponds with similar studies in Nigeria [14]. However, in another Nigerian study, that included nursing tutors from the university, a higher proportion of nurses (61.7%) had utilized research in practice [13]. Findings from this study, focusing primarily on direct care nurse, imply that majority of its participants keep working in accordance with yesterdays' instruction. The paradigm of EBNP can therefore not be said to be championed by these nurses.

**Barriers and socio-demographic factors influencing nurses participation in and utilisation of clinical research in practice**: The study revealed that insufficient time for nurses to read articles was associated with nurses' participation in research as it recorded a P-value of 0.04 at a 95% confidence interval. As studies from both Zimbabwe [17] and Singapore [18] show similar results, inadequate time may be a characteristic of nurses' work situation. Moreover, nurses' perception about research as not a nursing role was expressed in the qualitative interviews and also found to be statistically significant with a P-value of 0.04. This finding implies that many nurses interpret research to not be part of their professional profile, which could influence their motivation to read research articles even if time was available. In order to foster nurses' research participation and utilization, both measures are probably needed on both individual and institutional level. Based on the above findings, the null hypothesis of no statistical difference between characteristics of the nurse and participation in research was therefore rejected. As could be expected, educational qualification of nurses was found to be associated with participation in clinical research indicating that the higher the educational level the more the nurse is research-conscious. Similarly, studies in Nigeria identified that a significant relationship existed between nurses' educational qualification and their involvement in research after nursing education [13,19].

**Barriers and socio-demographic factors influencing utilization of research findings by nurses**: Findings from the study revealed that lack of cooperation from physicians and colleagues, perceived minimal benefit of research to clinical practice and nurses' lack of motivation for change were found to be statistically significant with P-values of 0.003, 0.01 and 0.02 respectively. As nurses, in this study, noted minimal experience from engaging in research and lack of institutional as well as collegial support for doing so, their low motivation for integrating research into practice is quite understandable. A similar studies [19,20] also documented that despite the fact that majority of the nurses had formal training in research methods, close to half held a unconvinced attitude towards the benefits of utilization of research in clinical practice. From the findings of this study, we therefore reject the null hypothesis of no statistical difference between characteristics of the setting and utilization of research. The qualitative interviews strengthens the tendency in the survey, as the key-informants too described nurses' unfamiliarity with principals for evidence based clinical practice as a barrier for their active engagement in implementing research findings. For nursing practice to be evidenced-based, nursing schools and regulatory bodies therefore need to design measures that continuously support the scientific and professional development of nurses' in clinical care.

## Conclusion

The study revealed that only 57 (36.1%) and 40 (25.3%) had ever participated and used research findings to change their practice after school respectively. Among the nurses who had ever participated in research, the majority did so as study participants and very few were principal investigators. Moreover, it was also found that insufficient time on the job to read research articles, perception about research as not part of the nursing role were associated with nurses participation in research. Among the socio-demographic factors considered for this study, nurses' educational qualification was found to be associated with participation in clinical research. Interviews with key-informants confirmed that inadequate time, lack of funding and nurses poor perception about research were barriers to research participation among nurses. Lack of cooperation from physicians and colleagues, perceived minimal benefit of research to practice and lack of motivation for change were factors associated with utilization of research in practice. However, none of the socio-demographic factors considered for the study was associated with utilization of research. The qualitative interviews also indicated a lack of appreciation for research among nurses as a barrier influencing their utilization of research.

### What is known about this topic

- Participation and utilization of research findings in practice by clinical nurses is low;
- Lack of time on the job, lack of funding, lack of knowledge and poor perception about research have been documented as the perceived barriers for nurses participation in clinical research;
- Lack of managerial support, lack of nurses' authority to change practice, results not generalizable to own setting and lack of time have frequently been documented as barriers influencing nurses participation and utilization of research findings.

### What this study adds

- Among the barriers, only nurses' perception about research as not a nursing role and insufficient time for nurses to read articles were found to be associated with nurses participation in clinical research;
- A perception by nurses about research not being part of the nursing role would negatively affect their enthusiasm to participate in studies which in turn affect professional development;
- Moreover, to help establish a positive research culture, hospital authorities should also reward nurses who conduct and incorporate research findings in their practice.

## Competing interests

Authors declare no competing interests.
